# Diffusion-weighted Renal MRI at 9.4 Tesla Using RARE to Improve Anatomical Integrity

**DOI:** 10.1038/s41598-019-56184-6

**Published:** 2019-12-23

**Authors:** Joāo dos Santos Periquito, Katharina Paul, Till Huelnhagen, Min-Chi Ku, Yiyi Ji, Kathleen Cantow, Thomas Gladytz, Dirk Grosenick, Bert Flemming, Erdmann Seeliger, Sonia Waiczies, Thoralf Niendorf, Andreas Pohlmann

**Affiliations:** 1 Berlin Ultrahigh Field Facility (B.U.F.F.), Max Delbrück Center for Molecular Medicine in the Helmholtz Association, Berlin, Germany; 2 Institute of Physiology, Charité Universitätsmedizin Berlin, Campus Mitte, and Center for Cardiovascular Research, Berlin, Germany; 3 Physikalisch-Technische-Bundesanstalt (PTB), Berlin, Germany; 4 Experimental and Clinical Research Center, a joint cooperation between the Charité Medical Faculty and the Max Delbrück Center for Molecular Medicine in the Helmholtz Association, Berlin, Germany; 5 German Centre for Cardiovascular Research (DZHK), partner site Berlin, Germany

**Keywords:** Preclinical research, Kidney, Biomedical engineering

## Abstract

Diffusion-weighted magnetic resonance imaging (DWI) is a non-invasive imaging technique sensitive to tissue water movement. By enabling a discrimination between tissue properties without the need of contrast agent administration, DWI is invaluable for probing tissue microstructure in kidney diseases. DWI studies commonly make use of single-shot Echo-Planar Imaging (ss-EPI) techniques that are prone to suffering from geometric distortion. The goal of the present study was to develop a robust DWI technique tailored for preclinical magnetic resonance imaging (MRI) studies that is free of distortion and sensitive to detect microstructural changes. Since fast spin-echo imaging techniques are less susceptible to B_0_ inhomogeneity related image distortions, we introduced a diffusion sensitization to a split-echo Rapid Acquisition with Relaxation Enhancement (RARE) technique for high field preclinical DWI at 9.4 T. Validation studies in standard liquids provided diffusion coefficients consistent with reported values from the literature. Split-echo RARE outperformed conventional ss-EPI, with ss-EPI showing a 3.5-times larger border displacement (2.60 vs. 0.75) and a 60% higher intra-subject variability (cortex = 74%, outer medulla = 62% and inner medulla = 44%). The anatomical integrity provided by the split-echo RARE DWI technique is an essential component of parametric imaging on the way towards robust renal tissue characterization, especially during kidney disease.

## Introduction

The prevalence of kidney diseases poses a major public health challenge. Acute kidney injury (AKI) is one of the leading causes of death with an estimated two million deaths per year. Chronic kidney disease (CKD) affects > 10% of the population and its incidence continues to grow^1–4^. There is still a critical need for translational approaches that study renal disease and renoprotective strategies^5–8^. The lack of these approaches underscores the importance of developing biomedical imaging techniques that probe different stages of kidney disorders to better understand the spatio-temporal changes of renal physiology in AKI or CKD and the underlying disease mechanisms^9,10^. It is becoming increasingly evident that quantitative MRI methods can provide vital biomarkers with respect to diagnosis, prognosis and disease prediction, as well as monitoring treatment response. Thereby MRI could improve acute and chronic renal disease management^11^ – either alone or as a complement to blood-, urine or tissue-based biomarkers^12^.

Diffusion-weighted MRI (DWI) allows quantitative measurements that reflect micro-morphological and physiological changes in renal tissues. Renal DWI requires further investigation before application to routine clinical use. Challenges that need to be overcome in order for research results of renal DWI to be translated to clinical practice, include the harmonization of the acquisition protocols, data post-processing and image analysis^11^. To facilitate the standardization and validation of renal DWI, consensus-based technical recommendations are currently being developed by an international, multidisciplinary group of renal imaging researchers as part of the European Cooperation in Science and Technology (COST) action ‘PARENCHIMA’ (www.renalmri.org). This is motivated by the relevance of renal DWI having been demonstrated in numerous preclinical and clinical studies with applications such as AKI^13^, characterization of renal masses^14^, tumors^15–18^, lesions^19,20^ and cysts^21^, as well as the assessment of renal fibrosis^22–25^, allograft pathophysiology^26,27^, diabetic nephropathy^28^, and functional changes in AKI and CKD^29,30^.

The most commonly employed DWI technique is single-shot echo planar imaging (ss-EPI) because of its fast imaging speed. It is currently the standard method on preclinical MR systems that is suitable for *in vivo* studies. It offers excellent temporal resolution, insensitivity to bulk motion and provides reasonable spatial resolution. Notwithstanding these advantages, ss-EPI is prone to magnetic susceptibility artifacts that present themselves as T_2_* induced signal loss or even signal voids in areas with very high B_0_ gradients, low-phase encoding bandwidth related image distortion and off-resonance effects caused by Δ*B*_0_ induced frequency dispersions^31^. Hence, diffusion weighted ss-EPI in kidney regions adjacent to bowels or in close proximity to skin/fat/muscle boundaries is particularly challenging and prone to loss of anatomical integrity due to geometric distortions.

Geometric distortions caused by ss-EPI may have serious consequences for the quantitative analysis of renal MRI data. Manual definition of regions-of-interest (ROI) can be extremely challenging when these image distortions are present, as it requires clear distinctions between the renal layers. Semi-automated ROI analysis techniques, such as concentric object analysis^32,33^ or the morphology-based ROI-placement^34,35^ can be severely compromised by geometric distortions.

Fast spin-echo imaging techniques are largely insensitive to B_0_ inhomogeneity related image distortions and hence present a valuable alternative to EPI particularly at (ultra)high magnetic field strengths^36–43^. This makes diffusion-sensitized fast spin-echo imaging a promising approach for improving anatomical integrity in renal DWI. The suitability of single-shot Rapid Acquisition with Relaxation Enhancement (ss-RARE^44^) for DWI has been shown for the human kidney at typical clinical field strengths of 1.5 T^45^ and 3.0 T^46^.

Motivated by the translational prospects of renal DWI along with the signal sensitivity gain at high magnetic fields, this work demonstrates the performance and reliability of a variant of diffusion-sensitized ultrafast RARE^36–44^ for DWI of the rodent kidney at 9.4 T. To meet this goal we implemented and adapted a diffusion-sensitized split-echo RARE (DW Split-echo RARE) technique^47^. First, we validated the proposed technique thoroughly in phantom studies. Then, we confirmed the hypothesis that DW Split-echo RARE outperforms the conventional DW ss-EPI in terms of anatomical integrity and variability of measurements in an *in vivo* DWI study in rats.

## Materials and Methods

### Implementation and optimization of the Split-echo RARE DWI technique

A Stejskal-Tanner preparation scheme was used to introduce diffusion sensitization to a RARE variant^48^: Diffusion gradients were placed around the first refocusing pulse and Split-echo RARE acquisition was implemented to avoid destructive interferences between even and odd echoes^47^. Further details on the pulse sequence (a pulse sequence diagram) are provided as Supplementary Information. Dummy RF pulses (n = 4) were applied prior to data acquisition to balance the signal amplitude between odd and even echo groups^49^. A central phase encoding scheme was employed to reduce the time between the diffusion sensitization module and the acquisition of the central k-space region. For comparison, the commonly used diffusion weighted spin-echo (SE) echo-planar imaging (EPI) method was used. A single-shot set-up was chosen, because of the excellent temporal resolution it provides, which is essential for *in vivo* studies of functional dynamics, such as those involving short and reversible physiological stimuli^50^ or the characterization of early pathophysiological events in AKI^34^.

### Phantoms

A phantom containing different substances with known diffusion properties was prepared in order to (i) validate the measured diffusion parameters and to (ii) examine the propensity of DW ss-EPI and DW Split-echo RARE to geometric distortions. The custom-made phantom consisted of three tubes (outer diameter: 7.8 mm) filled with vegetable oil (sunflower oil), de-ionized water, and acetone, respectively. These tubes were placed in a larger cylindrical tube (outer diameter: 30 mm) filled with a 5% solution of agarose to facilitate the imaging, achieve a sensible loading of the RF coil, and reduce macroscopic distortions of the magnetic field B_0_.

### Animals

All investigations were approved by the Animal Welfare Department of Berlin’s State Office of Health and Social Affairs (*LaGeSo*) in accordance with the German Animal Protection Law. The procuration of animals, husbandry and experiments conformed to the European Convention for the Protection of Vertebrate Animals used for Experimental and other Scientific Purposes (Council of Europe No 123, Strasbourg 1985). The animals had ad libitum access to food (standard diet) and water and were housed under standard conditions with environmental enrichment. Female Wistar rats (aged 12–13 weeks, body weight 288–330 g, n = 7; Harlan-Winkelmann, Borchen, Germany) underwent MRI under isoflurane anesthesia (2.0% in air). All animals scanned were included in the data analysis. Core body temperature was monitored by means of a rectal fiber-optic temperature probe (AccuSens, Opsens, Québec City, Canada). Body temperature was maintained at 37 °C with a pad containing circulating warm water connected to a water-bath. Respiration rate was monitored throughout the experiment using a small balloon placed on the chest of the animal (Model 1025, SA Instruments, Stony Brook, NJ, USA) and served for triggering the MRI data acquisition.

### Magnetic resonance imaging

All MRI measurements were carried out on a 9.4 Tesla small animal MR system (Bruker Biospec 94/20; Bruker Biospin, Ettlingen, Germany). For phantom experiments, a quadrature transceiver birdcage radiofrequency (RF) volume resonator (inner diameter: 72 mm; Bruker Biospin, Ettlingen, Germany) was employed. *In vivo* studies used a curved 4-channel surface RF coil array (rat heart RF coil, Bruker Biospin, Ettlingen, Germany) for signal reception in conjunction with the above birdcage volume resonator for signal transmission.

We compared the proposed DW Split-echo RARE approach against the DW spin-echo EPI method commonly used in rodents. Images obtained from a DW spin-echo (SE) sequence were used as reference for the phantom assessments. *In vivo*, a gradient-echo sequence (FLASH) served as anatomical reference to assess geometric distortions, since the very long acquisition time of DW SE imaging render this method unfeasible for *in vivo* MRI. These comparisons aimed to validate the measured diffusion parameters and also to examine the propensity of DW ss-EPI and DW Split-echo RARE for geometric distortions.

DWI was performed on the phantom and on seven Wistar rats *in vivo* using the imaging parameters summarized in Table 1. Acquisition parameters were chosen such that both DW ss-EPI and DW Split-echo RARE had the same acquisition time. Apart from the fast single-shot protocols, a multi-shot protocol was also used for DW Split-echo RARE to demonstrate the image quality achievable at higher spatial resolution. Such protocols for diffusion sensitized Split-echo RARE kidney MRI would be of interest for experiments in which high temporal resolution is not essential, such as in chronic kidney disease (CKD) where pathological changes are rather sluggish.

**Table 1:** Summary of DWI acquisition parameters for the three set-ups used in the experiments.

Experimental set-up	(1) phantom, (2) *in vivo*		(3) *in vivo* (high-resolution)
DWI technique	ss-EPI	Split-echo RARE	Split-echo RARE
Receiver Bandwidth	300 kHz	131 kHz	131 kHz
Echo time (TE)	25 ms	16 ms	20 ms
Repetition time (TR)	approx. 2.3 s (respiration triggered)		
Field of view	(45 × 45) mm^2^		
Effective Acquisition matrix	96 × 192		128 × 256
Spatial resolution	0.47 × 0.23		0.35 × 0.18
Slice thickness	1.5 mm		1.5 mm
segments/Echo train length (ETL)	1/192		4/64
Averages	8		8
Acquisition time per b-value	18 s		73 s
Total acquisition time (5 × 3 b-values)	4 min 30 s		18 min 15 s

Diffusion weighting was achieved using b-values of 0, 200, 300, 500 and 700 s/mm^2^. In the homogenous phantom one diffusion direction was used. To account for diffusion anisotropy effects in the kidney, each of these acquisitions was performed in three orthogonal diffusion sensitization directions, yielding 15 (5 b-values × 3 directions) acquisitions in total. Respiratory motion artifacts were reduced by triggering the data acquisition based on the respiratory signal trace.

### Quantification of water diffusion

#### Phantom

Diffusion coefficient maps were generated for the diffusion phantom by a pixel-wise linear fitting performed after taking the logarithm of the signal intensities obtained at the 5 b-values.

1 $${\rm{S}}({\rm{b}})={{\rm{S}}}_{0}.{{\rm{e}}}^{-{\rm{bD}}}$$

where *S* is the signal intensity and *S*_0_ is defined as the signal intensity at b = 0 s/mm^2^. *D* is the diffusion coefficient also referred to as the ‘apparent diffusion coefficient’ (ADC), recognizing that is depends on both, the nature of the media studied and on experimental conditions. ROI analysis was performed to determine the mean diffusion coefficient for the three compounds, which were benchmarked against literature values.

#### Rat kidney *in vivo*

The intra-voxel incoherent motion (IVIM) approach was used. This consists of a two compartment bi-exponential model, in order to obtain pure diffusion values, without contamination from pseudo-diffusion (i.e. incoherent movement of water by blood perfusion). According to the IVIM approach, the relation between signal intensity and the b-values can be described as:

2 $${\rm{S}}({\rm{b}})={{\rm{S}}}_{0}({{\rm{fe}}}^{-{{\rm{bD}}}_{{\rm{p}}}}+(1-{\rm{f}}){{\rm{e}}}^{-{\rm{bD}}})$$

where *S* is the signal intensity, *D*_*p*_ is pseudo-diffusion coefficient, *f* is flow fraction and *D* is slow diffusion (pure diffusion) coefficient. *S*_0_ is defined as the signal intensity at b = 0 s/mm^2^. For b ≥ 200 s/mm^2^ no contribution from *D*_*p*_ is assumed because the signal decay of *D*_*p*_ is much faster than *D* (*D*_*p*_ ≫ *D*). Therefore, we calculated the pure diffusion coefficient from a non-linear least square fit to the signal intensities at b ≥ 200 s/mm^2^, which allowed us to use the simplified Eq. 1. All three directions were averaged to account for diffusion anisotropy.

ROIs were defined according to the morphological features of the kidney using semi-automated kidney segmentation^35^. ROIs were defined on a coronal kidney image: 5 in the renal cortex (COR), 5 in the outer medulla (OM) and 3 in the inner medulla (IM) as previously described^35^. The mean diffusion coefficient of each renal layer (COR, OM, IM) was computed as the average of all ROIs within the layer.

### Quantification of geometric distortion

Contours drawn around the cylindrical structures of the phantom in the spin-echo (DW SE) images were defined as the distortion-free reference. For *in vivo* DWI, a contour drawn around the kidney on a gradient-echo image was used instead. These contours were determined by two experienced MR experts, under the supervision of a senior board certified radiologist. To illustrate the extent of geometric distortions in DW ss-EPI and DW Split-echo RARE, we used color-coded difference-maps between the contours drawn in these images and the reference contour.

**Figure 1:**
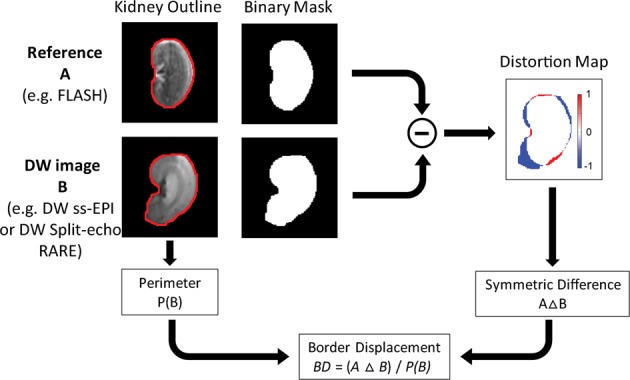
Assessing geometric distortion. The kidney outline (red contour - border of the kidney) was created for the reference image and the DW image. Binary masks were generated for each image using the kidney outline. The distortions maps were calculated by subtracting the binary mask of the DW image from the binary mask of the reference image, a pseudo-colour scale [−1 1] was used from blue (−1) to red (+1). The red pixels of the distortion map represent false negatives, and the blue pixels represent false positives, for the DW image with respect to the reference mask. Border displacement was calculated by dividing the number of pixels of the distortion map (*A Δ B*) by the perimeter (*P(B)*) of the kidney outline of the DW Image.

**Figure 2:**
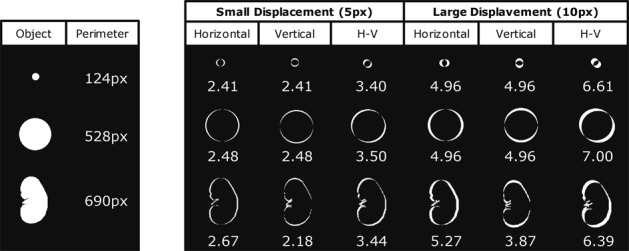
The border displacement metric is fairly insensitive to the object’s size and shape. Horizontal, vertical and diagonal displacements (5 or 10 pixels) were artificially applied to three objects of different shape and size: a small circle (top row), a large circle (middle row) and a kidney shape (bottom row). The border displacement (BD) is given below each object. While the simulated small and large displacements lead to similarly different BD (compare left and right), the BD varied only little between the three different objects (compare along vertical axes).

Geometric distortions were quantified using an in-house developed method of border displacement analysis (Fig. 1; program written in MATLAB; The Mathworks, Natick, MA, USA). The metric *border displacement* between two contours was based on their *symmetric difference*, an elementary mathematical operation of set theory. The *symmetric difference* of two sets, *A* and *B*, includes all objects (here the pixels) in A and B (here the two contours) that are outside their intersection: *A* Δ *B* := {*x* | (*x* ∈ *A* Λ *x* ∉ *B*) ∨ (*x* ∈ *B* Λ *x* ∉ *A*)}. For the simple case of two identical contours, there are no pixels outside their intersection, hence the symmetric difference is zero. The greater the geometric difference between contours *A* and *B*, the larger the number of pixels outside their intersection, i.e. the larger their symmetric difference. To permit meaningful comparisons of *border displacements* calculated from images with different spatial resolution, and even from objects of different shape and size (e.g. tubes, organs or other structures) we normalized the symmetric difference to the contour length, i.e. the object’s perimeter *P*, and defined the *border displacement* (*BD*) as

3 $$BD=(A\,\Delta \,B)/P(B)$$

with *A* being the contour to be assessed, *B* the reference contour, and *P(B)* the perimeter of the reference contour; or verbose: *Border displacement* = *Symmetric difference*(*Contour, Reference-contour*)/*Perimeter(Reference-contour)*. This metric yields comparable results for the same extent of distortion applied to objects of different sizes and shapes, as demonstrated with the simulations shown in Fig. 2.

### Statistical tests

A normal distribution of the calculated border displacements and diffusion coefficients cannot be assumed, because the susceptibility-induced image distortions influence both in an unpredictable manner. To this end, the results are given as the median together with the minimum to maximum value range (rather than the commonly used mean ± standard deviation). For testing statistical differences we used the non-parametric Mann-Whitney U test with a significance level of 5%. This test does not make any assumptions about the form of the distributions, requiring only that both groups have the same distributions under the null hypothesis.

## Results

We acquired experimental data to validate that the diffusion parameters measured with the DW Split-echo RARE are correct, to assess the geometric fidelity of the images, and to detail the diffusion parameters obtained in healthy rat kidneys.

### Diffusion measurements

**Figure 3:**
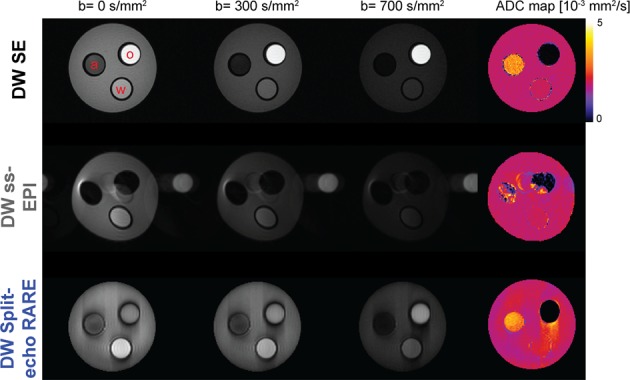
Diffusion weighted images and derived diffusion maps of the test phantom. Selection of images with b-values in the range b = 0 s/mm^2^ to b = 700 s/mm^2^ acquired with DW SE (top row), DW ss-EPI (middle row) and DW Split-echo RARE (bottom row). The right column shows corresponding diffusion maps for each of the three approaches calculated from the series of diffusion-weighted images, a pseudo-color scale [0 5] was used from black (0) via purple, red, orange, yellow to white (5). The cylindrical phantom contains acetone (“a”), water (“w”) and vegetable oil (“o”) in three tubes within a larger tube with agarose. As expected, one can observe the inherent geometric distortions with DW ss-EPI (due to the low effective bandwidth in phase encoding direction) and blurring with DW Split-echo RARE (due to the effect of the T_2_-decay on the point-spread-function and inter-echo spacing). The artifacts along the phase encoding direction obtained for DW split-echo RARE are due to the very sharp boundaries and strong signal intensity changes in the phantom and the length of the echo train (192 × 3.2 ms).

**Figure 4:**
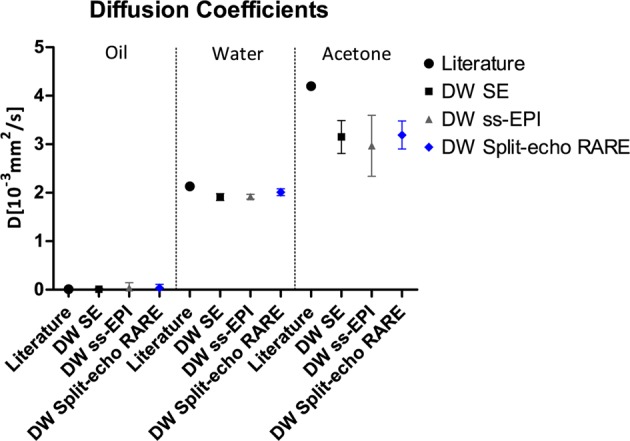
Validation of measured diffusion coefficients for a phantom with known diffusion properties. The diffusion coefficient for vegetable oil, water, and acetone were measured using three diffusion-weighted acquisition techniques: spin-echo (SE; reference, 0.011 ± 0.019, 1.91 ± 0.07 and 3.15 ± 0.34 10^−3^ mm^2^/s), DW ss-EPI (0.041 ± 0.10, 1.92 ± 0.05 and 2.97 ± 0.63 10^−3^ mm^2^/s), and DW Split-echo RARE (0.035 ± 0.074, 2.01 ± 0.07 and 3.19 ± 0.29 10^−3^ mm^2^/s). Results are compared against literature diffusion coefficients values of vegetable oil (0.010 10^−3^ mm^2^/s)^67^ water (2.13 10^−3^ mm^2^/s)^68^ and acetone (4.21 10^−3^ mm^2^/s)^69,70^. Diffusion coefficient are in units of 10^−3^ mm^2^/s (mean over ROIs ± standard deviation) for ROIs placed in the diffusion coefficient maps for each respective material.

To validate the diffusion weighting of the Split-echo RARE, phantom experiments were conducted at room temperature using a cylindrical phantom containing 3 tubes each loaded with water, vegetable oil and acetone. Figure 3 illustrates diffusion-weighted images for selected b-values and the corresponding parameter maps of the diffusion coefficient (D) calculated for DW SE reference acquisitions as well as DW Split-echo RARE and DW ss-EPI measurements. For a quantitative comparison, the diffusion coefficients derived from these measurements are shown in Fig. 4, together with the literature values. We observed a good agreement between all three DW approaches. However, the DW Split-echo RARE method resulted in diffusion coefficients (for water and acetone) that were closer to the literature values than those obtained with the DW ss-EPI and even reference DW SE method.

### Geometric fidelity assessment of DWI in phantom experiments

**Figure 5:**
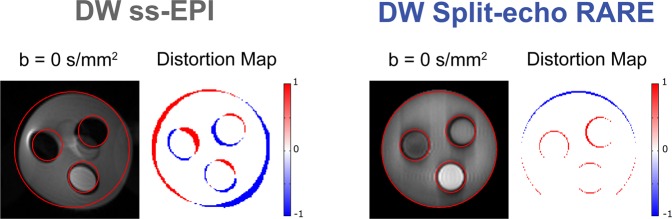
Assessment of geometric fidelity in the test phantom. Images obtained for a structured phantom consisting of three small cylinders within one large one at 9.4 T using DW ss-EPI (left panel) and DW Split-echo RARE (right panel). The overlaid red contour represents the geometry of the DW SE reference image, which was acquired with the same spatial resolution and matrix size. The color-coded difference maps with respect to the DW SE reference visually demonstrate the amount of distortion, a pseudo-color scale [−1 1] was used from blue (−1) via white to red (1). The artifacts along the phase encoding direction obtained for DW Split-echo RARE are due to the very sharp boundaries and strong signal intensity changes in the phantom and the length of the echo train (192 × 3.2 ms).

To examine the geometric fidelity of the DWI approaches, the border displacement (BD) analysis was applied to the phantom images of DW Split-echo RARE and DW ss-EPI, without diffusion weighting (b = 0 s/mm^2^) (Fig. 5). Red contours derived from the DW SE reference image were superimposed onto the DW Split-echo RARE and DW ss-EPI images (Fig. 5). Difference (distortion) maps showed that DW Split-echo RARE yields close to distortion-free images at 9.4 T with a border displacement of 0.50 [0.31; 0.73] (median of 4 circles [minimum; maximum]. On the other hand, pronounced displacements 1.87 [1.37; 2.41] were observed for DW ss-EPI.

### Anatomical integrity assessment of *in vivo* renal DWI

**Figure 6:**
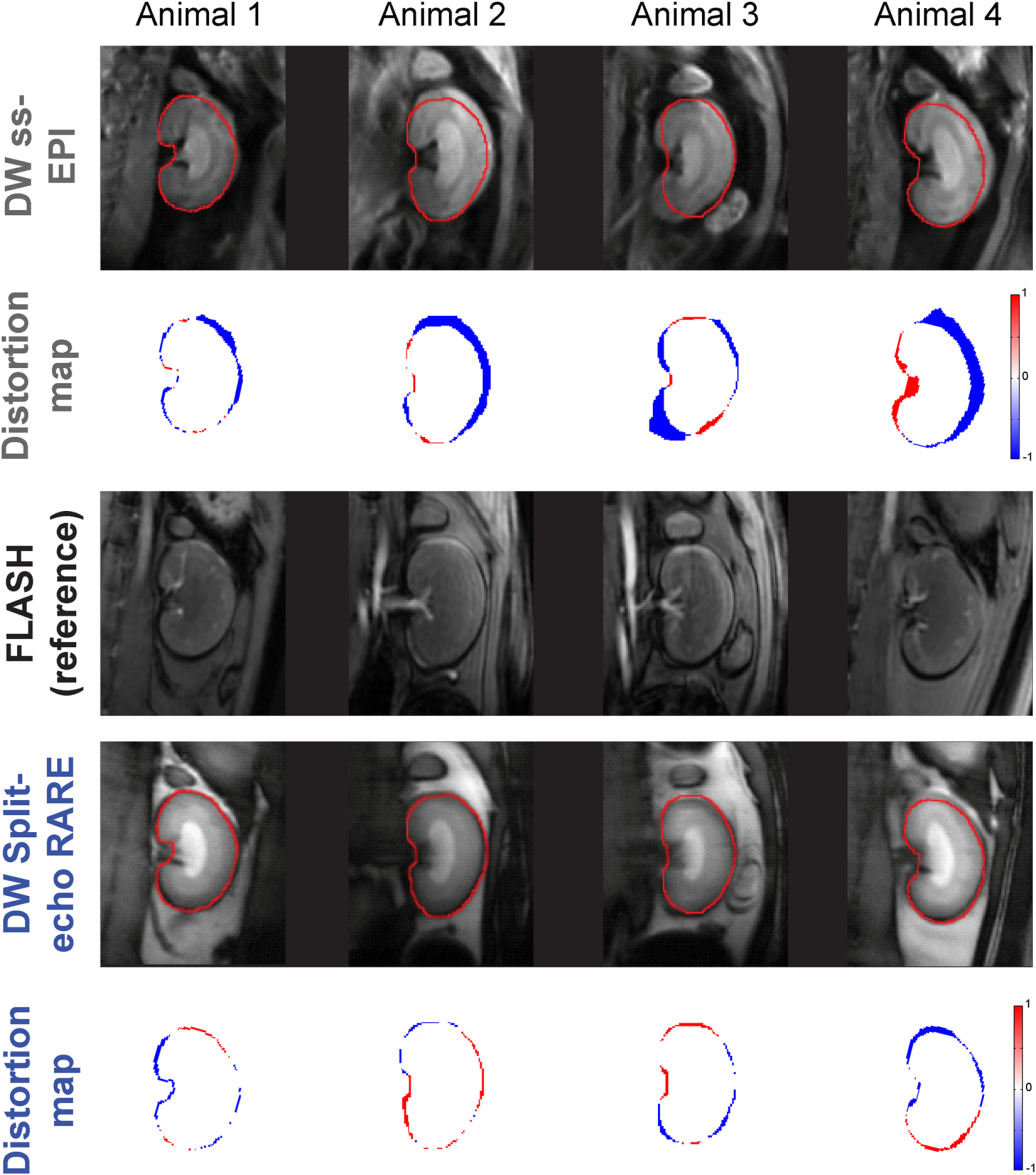
Assessment of geometric distortions *in vivo*. Coronal images of rat kidneys obtained in seven animals *in vivo* at 9.4 T using, DW ss-EPI (b = 0 mm/s^2^) (top row), FLASH (third row) and DW Split-echo RARE (b = 0 mm/s^2^) (fourth row). The respective distortion map (compared to the FLASH reference) is shown below each MR image (third and fifth row), a pseudo-color scale [−1 1] was used from blue (−1) via white to red (1). The red contour represents the border of the kidney in the FLASH reference images, which have high geometric fidelity. Border displacement was markedly smaller with DW Split-echo RARE than with DW ss-EPI.

**Figure 7:**
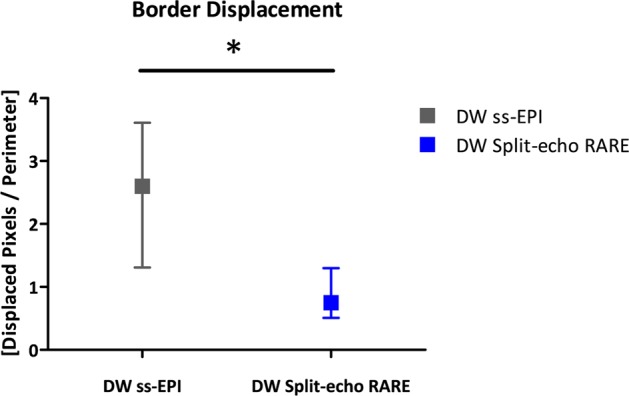
Quantification of border displacement analysis and comparison between DW methods. The median of the measured border displacement (arbitrary units) for DW ss-EPI was significantly larger (p < 0.05) than that for the proposed DW Split-echo RARE approach, the error bars indicate minimum and maximum value.

To access anatomical distortions *in vivo*, coronal slices of the rat kidney were acquired using ss-EPI and Split-echo RARE, as well as FLASH (as an anatomical reference). Similar to the phantom experiments, Split-echo RARE provided almost distortion-free images at 9.4 T as demonstrated by the distortion maps (Fig. 6). Border displacement analysis yielded BD = 0.75 [0.51; 1.30] pixels for Split-echo RARE. In contrast, the border displacement for ss-EPI was significantly (p = 0.0006) higher: 2.60 [1.31; 3.61] pixels (Fig. 7).

### Renal *in vivo* diffusion measurements

**Figure 8:**
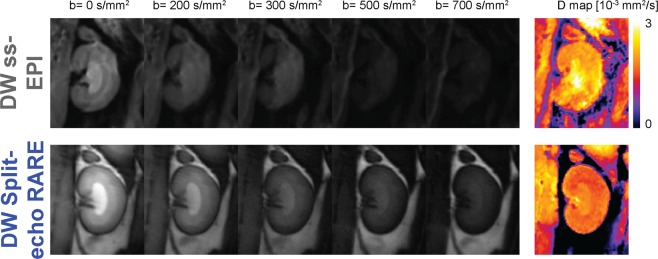
IVIM images examples and corresponding diffusion maps. Images of a rat kidney acquired at 9.4 T with diffusion weightings raging from (0 to 700 s/mm^2^) using the conventional DW ss-EPI (top row) or the DW Split-echo RARE (bottom row) method within a total acquisition time of 4.5 minutes. Respective parameter maps of the diffusion coefficient are shown in the right column, a pseudo-color scale [0 3] was used from black (0) via purple, red, orange, yellow to white (3). The quality of the MR images and parameter map was markedly better with DW Split-echo RARE compared with DW ss-EPI in all seven animals.

**Figure 9:**
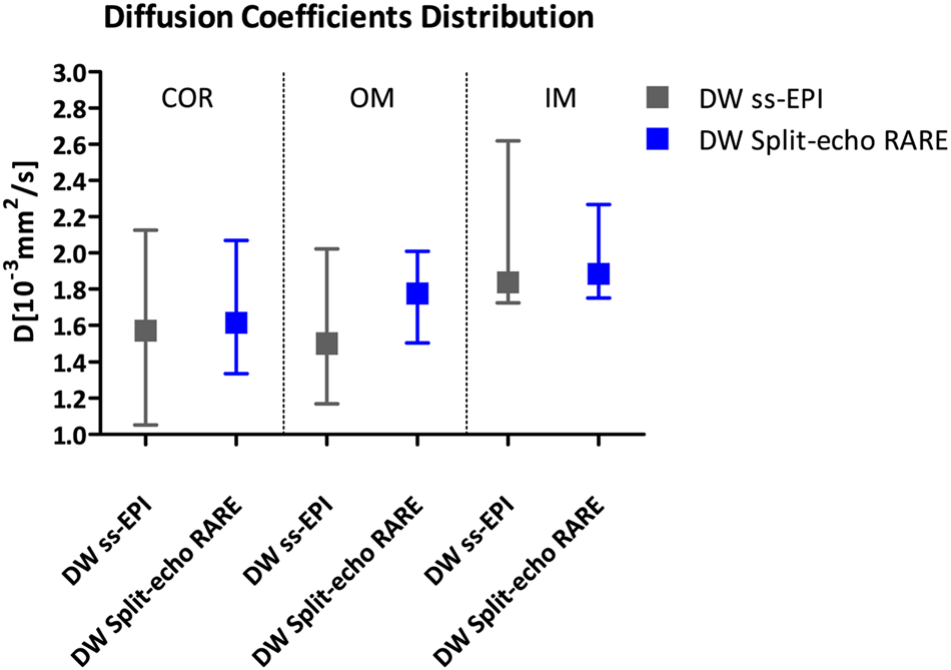
Comparison of D for the renal cortex (COR), outer medulla (OM) and inner medulla (IM) and comparison between DW methods. Data were obtained from seven rats. D median (using DW ss-EPI (grey), D median using DW Split-echo RARE (blue) for each different region on the kidney: COR, OM and IM. The error bars indicate minimum and maximum value. DW Split-echo RARE yielded for all renal compartments a smaller [minimum; maximum] range than the obtained with DW ss-EPI.

The *in vivo* MR images (with varying b values) and corresponding parameter maps obtained with the Split-echo RARE method were of markedly better quality and showed fewer artefacts (e.g. susceptibility distortion) than those obtained with the ss-EPI (Fig. 8). Renal IVIM analysis of the *in vivo* DW Split-echo RARE data from seven animals yielded a diffusion coefficient of *D*_COR_ = 1.61 [1.34; 2.07] × 10^−3^ mm^2^/s for the renal cortex, *D*_OM_ = 1.78 [1.50; 2.01] × 10^−3^ mm^2^/s for the outer medulla and *D*_IM_ = 1.88 [1.75; 2.27] × 10^−3^ mm^2^/s for the inner medulla. The results obtained with DW ss-EPI were similar, but varied much more between subjects (Fig. 9), as is evident from the larger difference of [minimum, maximum]: *D*_COR_ = 1.57 [1.05; 2.13] × 10^−3^ mm^2^/s, *D*_OM_ = 1.50 [1.17; 2.02] × 10^−3^ mm^2^/s and *D*_IM_ = 1.84 [1.72; 2.62] × 10^−3^ mm^2^/s.

#### Intra-renal variability and variance

**Figure 10:**
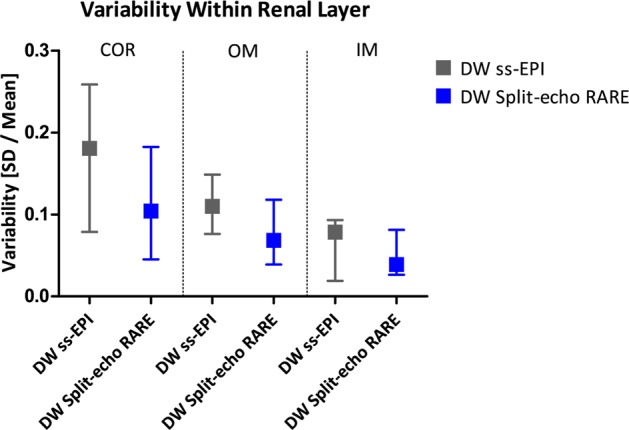
Diffusion variability within each renal layer. Variability [SD/Mean] within the different layers, i.e. cortex (COR), outer medulla (OM), and inner medulla (IM) was calculated as a surrogate of measurement quality. Variability median (using DW ss-EPI (grey), Variability median using DW Split-echo RARE (blue) for each different region on the kidney: COR, OM and IM. The error bars indicate minimum and maximum value. DW Split-echo RARE yielded for all renal compartments a smaller median and [minimum; maximum] range than the obtained with DW ss-EPI.

The variability (calculated as SD/mean) and variance (SD^2^) of *D* within each renal layer were calculated as surrogates of measurement quality, based on the assumption that in a healthy kidney the water diffusion properties are similar within the tissue of a given renal layer. The plot of intra-renal diffusion variability in Fig. 10 demonstrates a smaller variability for DW Split-echo RARE (cortex: 0.104 [0.045; 0.182], outer medulla: 0.068 [0.038; 0.118], inner medulla: 0.039 [0.026; 0.081] than for DW ss-EPI (cortex: 0.181 [0.079; 0.259], outer medulla: 0.110 [0.076; 0.149], inner medulla: 0.078 [0.019; 0.093]. This effect is based on the also smaller variance for DW Split-echo RARE (cortex: 0.015, outer medulla: 0.016, inner medulla: 0.005 than for DW ss-EPI (cortex: 0.050, outer medulla: 0.025, inner medulla: 0.020).

#### High spatial resolution DWI

**Figure 11:**
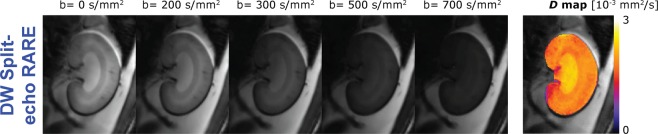
High-spatial resolution IVIM images and diffusion map. DW Split-echo RARE images of a rat kidney acquired *in vivo* at 9.4 T using the high spatial resolution protocol and diffusion weightings of b = 0 to 700 s/mm^2^, together with the respective *D* map, a pseudo-color scale [0 3] was used from black (0) via purple, red, orange, yellow to white (3). The excellent image/map quality obtained with this 18-minute scan makes the DW Split-echo RARE approach attractive for renal steady-state MRI experiments, such as in CKD models.

The high resolution protocol supported an in-plane spatial resolution of (0.35 × 0.18) mm^2^ and provided excellent image quality (Fig. 11), which revealed the more subtle structures of the cortico-medullary transition with better contrast.

## Discussion

In this work we demonstrate the feasibility of DW Split-echo RARE for renal diffusion weighted imaging (DWI) in small rodents at 9.4 T. We confirmed our hypothesis that DW Split-echo RARE outperforms the conventional DW ss-EPI method in terms of geometrical/anatomical integrity and measurement variability, especially in the *in vivo* experiments.

IVIM analysis of the DW Split-echo RARE data yielded mean diffusion coefficients of 1.65 × 10^−3^ mm^2^/s in the cortex, 1.75 × 10^−3^ mm^2^/s in the outer medulla, and 1.96 × 10^−3^ mm^2^/s in the inner medulla. These values are consistent with those reported in the literature when using DW-EPI^51–53^. Results obtained from ss-EPI images were similar to those derived from DW Split-echo RARE but showed a larger variability, especially in the outer and inner medulla. This is probably due to the image artifacts that are observed with ss-EPI. The lower variability and variance in the diffusion coefficients increases the effect size (standardized mean difference between two groups) and hence the statistical power to detect small pathophysiological changes, e.g., in x-ray contrast medium-induced AKI, or during initial stages of diabetic kidney disease.

Anatomical integrity was excellent for the Split-echo RARE technique, and far superior to the ss-EPI approach, which resulted in 3.5-fold larger border displacements for ss-EPI. Severe geometric distortions in the presence of magnet field inhomogeneities are expected with EPI, due to its low effective readout bandwidth in phase encoding direction. Echo-planar imaging variants are commonly used for renal DWI but are prone to magnetic susceptibility artifacts induced by the air-filled bowels, cavities and tissue interfaces surrounding the kidneys. Susceptibility artifacts compromise the anatomical integrity of DWI EPI kidney images and are even prevalent at lower magnetic field strengths^46,54,55^. Typically, images with severe distortions that cannot be corrected have to be eliminated from analyses. Due to complexity of non-linear geometric distortions, it is conceivable that such distortions might introduce errors when using (semi)automated analysis techniques that assume a certain kidney morphology (e.g. the morphology-based placement of ROIs^34,35^ or the Twelve-Layer Concentric Objects (TLCO) technique^33^). Yet, these detrimental effects may be reduced when large ROIs are used. Taken together, anatomical distortions can lead to increased variability and even unusable data, which in turn hamper intra- and inter-subject comparisons, and may compromise the statistical power of group analyses. Hence, keeping a good anatomical integrity in DWI is critical in order to achieve reliable results in the healthy as well as diseased kidneys – this is the forte of spin-echo-based techniques such as RARE.

Strategies for reducing geometric distortions resulting from predominantly gradient-echo-based EPI include image registration/unwarping. However, their practical use is somewhat limited, as outlined in the following. Registration of images containing geometric distortions is challenging, since it requires good image contrast and high degrees of freedom. In principle, unwarping (correction of susceptibility-induced distortions) can be done when additional field maps are acquired, however this is not trivial for strongly warped regions that lack a unique solution. Furthermore, unwarping will not be accurate if the distortions change during the relatively long DWI scan.

Segmentation of data collection, i.e. multi-shot acquisition, is another approach to reduce geometric distortions. Segmentation in phase-encoding direction can be applied in a common DW EPI protocol, but segmentation in readout direction requires a dedicated sequence. Readout segmented EPI (rs-EPI) in association with parallel imaging has been shown to reduce susceptibility artifacts in DWI^31,56–59^, improve renal DWI quality^55^, and correlate better with renal fibrosis^54^. One major drawback of segmented acquisitions is the associated longer acquisition times. Dividing the acquisition into 2 (or 4) segments already doubles (or quadruples) the acquisition duration. Increasing scan time for DWI — that already requires repeated acquisitions for different diffusion weightings and directions — represents a limitation in preclinical studies of physiological dynamics and in clinical routine use. Motion between the acquisitions of different segments in phase-encoding direction can create substantial artifacts. To diminish the shot-dependent nonlinear phase differences that arise from non-rigid motion, navigator readout segments need to be acquired repeatedly throughout the scan. Even though one could argue that the image quality of both, EPI and RARE, benefits significantly from segmentation, and hence multi-shot RARE suffers from the same time-constraints as rs-EPI, we have previously shown that even readout-segmented-EPI is less effective than ultrafast unsegmented RARE approaches for DWI in terms of restoring anatomical integrity^60^.

The use of refocusing pulses in RARE comes with an increase in the inter-echo time resulting in an increase in the echo train length versus EPI. Therefore the echo-train length used in RARE should receive attention when designing the imaging protocol and should not substantially exceed the T_2_ relaxation time of the object under investigation to avoid smearing artifacts along the phase encoding direction which may otherwise impair the ADC assessment. Imaging speed and RF power deposition are also recognized limitations of RARE when compared to EPI. To offset these constraints, Combined Acquisition Techniques (CAT)^61^ has been applied to boost imaging speed and reduce RF power deposition by using a modular hybrid approach that integrates a minimum of two imaging strategies. RARE-EPI CAT hybrids have been implemented for abdominal imaging^62^ and could hold the promise to further improve renal DWI at (ultra)high fields by combining of advantages of RARE (anatomical integrity) with those of EPI (imaging speed and less RF power deposition). The proposed Split-echo RARE approach is compatible with segmented acquisitions. To address potential motion induced phase changes, Split-echo RARE can be combined with the navigator echo approach^31^. To further enhance imaging speed, single shot and segmented Split-echo RARE can be supported by multiband RF pulses facilitating simultaneous multi-slice imaging. A recently developed multiplexed sensitivity encoding approach^63^ is also compatible with Split-echo RARE and affords reconstruction of multi-shot DWI data without the need of navigator echoes.

In our present study we made an experimental comparison between single-shot EPI and RARE protocols using the same short acquisition times. A thorough comparison between both fast protocols is critical for dynamic preclinical studies and when translating DWI into clinical situations that are highly dependent on imaging speed. In scenarios where temporal resolution is less crucial, a segmented approach may be introduced to (i) reduce the point spread function related blurring observed when using RARE with high echo-train-lengths (due to the effect of the T_2_-decay on the point-spread-function and the inter-echo time), and (ii) reduce geometric distortions in EPI but may also require additional navigator-based motion correction.

The current Split-echo RARE implementation uses a pair of unipolar Stejskal-Tanner gradients^48^, which helps to balance diffusion sensitization time and diffusion sensitization strength. However, pairs of unipolar gradients are not motion compensated and can be prone to eddy current related artifacts. Pairs of bipolar diffusion sensitizing gradients or twice-refocused gradients offer those features but come with a less effective sensitization^40,64,65^. Here, we chose a Split-echo approach using an imbalance in the readout gradient in order to preserve signal-to-noise ratio (SNR). With the Split-echo approach the spatial resolution along the read-out direction is only half of the displaced echo approach, which presents an alternative for avoiding interferences between odd and even echo groups^36,41^.

To conclude, this study demonstrates that Split-echo RARE has the capability to acquire distortion-free diffusion-weighted images of the rat kidney at ultrahigh magnetic field strengths. Improving anatomical integrity in DWI is a further step towards advancing the capabilities and robustness of parametric imaging of the kidney. It facilitates the use of semi-automated analysis methods that place ROIs reproducibly at clearly defined locations within the kidney based on kidney border information^33–35^. Identification of the kidney boundary is usually done manually^33^, which inevitably adds inter-observer variability to the results. A very valuable refinement to renal DWI would be the use of automated kidney segmentation methods such as the appearance-guided deformable boundary technique, which was recently shown to perform better than several alternative methods^66^. All these (semi-)automated approaches are important directions for further developments, as they help to eliminate any bias introduced by subjective manual interaction.

Adding robust DWI protocols to other MRI-based methods of tissue characterization of the kidney will further assist the non-invasive interrogation and phenotyping of small rodents during physiological interventions or pathological scenarios. Ultimately, the translational approach of the proposed Split-echo RARE method may be exploited for detecting and quantifying early renal disease in patients as well as studying disease mechanisms and renoprotective strategies in the future.

## Supplementary information

Supplementary Information

## References

[1] Zuk A, Bonventre JV (2016). Acute kidney injury. Annual review of medicine.

[2] Thakar CV, Christianson A, Freyberg R, Almenoff P, Render ML (2009). Incidence and outcomes of acute kidney injury in intensive care units: a Veterans Administration study. Critical care medicine.

[3] Thakar CV (2013). Perioperative acute kidney injury. Advances in chronic kidney disease.

[4] Ali T (2007). Incidence and outcomes in acute kidney injury: a comprehensive population-based study. Journal of the American Society of Nephrology: JASN.

[5] Zarjou A, Sanders PW, Mehta RL, Agarwal A (2012). Enabling innovative translational research in acute kidney injury. Clinical and translational science.

[6] Evans RG, O’Connor PM (2013). Initiation and progression of chronic kidney disease: can we definitively test the chronic hypoxia hypothesis?. Hypertension.

[7] Persson PB (2017). Renoprotection. Acta Physiologica.

[8] Matejovic M (2016). Renal Hemodynamics in AKI: In Search of New Treatment Targets. Journal of the American Society of Nephrology: JASN.

[9] Khwaja A (2012). KDIGO clinical practice guidelines for acute kidney injury. Nephron. Clinical practice.

[10] Molitoris BA (2015). Urinary Biomarkers: Alone Are They Enough?. Journal of the American Society of Nephrology: JASN.

[11] Selby Nicholas M, Blankestijn Peter J, Boor Peter, Combe Christian, Eckardt Kai-Uwe, Eikefjord Eli, Garcia-Fernandez Nuria, Golay Xavier, Gordon Isky, Grenier Nicolas, Hockings Paul D, Jensen Jens D, Joles Jaap A, Kalra Philip A, Krämer Bernhard K, Mark Patrick B, Mendichovszky Iosif A, Nikolic Olivera, Odudu Aghogho, Ong Albert C M, Ortiz Alberto, Pruijm Menno, Remuzzi Giuseppe, Rørvik Jarle, de Seigneux Sophie, Simms Roslyn J, Slatinska Janka, Summers Paul, Taal Maarten W, Thoeny Harriet C, Vallée Jean-Paul, Wolf Marcos, Caroli Anna, Sourbron Steven (2018). Magnetic resonance imaging biomarkers for chronic kidney disease: a position paper from the European Cooperation in Science and Technology Action PARENCHIMA. Nephrology Dialysis Transplantation.

[12] Abdeltawab H (2019). A Novel CNN-Based CAD system for early Assessment of transplanted Kidney Dysfunction. Scientific reports.

[13] Hueper, K. *et al*. T2 Relaxation Time and Apparent Diffusion Coefficient for Noninvasive Assessment of Renal Pathology After Acute Kidney Injury in Mice Comparison With Histopathology. 48 (2013). 10.1097/RLI.0b013e31829d0414 23907103

[14] Kang SK (2015). DWI for renal mass characterization: Systematic review and meta-analysis of diagnostic test performance. American Journal of Roentgenology.

[15] Aslan M (2017). Diffusion-weighted MRI for differentiating Wilms tumor from neuroblastoma. Diagnostic and Interventional Radiology.

[16] Lei Y (2015). Diagnostic Significance of Diffusion-Weighted MRI in Renal Cancer. BioMed Research International.

[17] Zhang, W. *et al*. HHS Public Access. 133, 48–61, 10.1161/CIRCULATIONAHA.115.017472.Critical (2017).

[18] Zhu Q (2019). Value of intravoxel incoherent motion for differential diagnosis of renal tumors. Acta Radiol.

[19] Attariwala R, Picker W (2013). Whole body MRI: Improved lesion detection and characterization with diffusion weighted techniques. Journal of Magnetic Resonance Imaging.

[20] Zhang YL (2013). EADC Values in Diagnosis of Renal Lesions by 3.0 T Diffusion-Weighted Magnetic Resonance Imaging: Compared with the ADC Values. Applied Magnetic Resonance.

[21] Franke M (2017). Magnetic resonance T2 mapping and diffusion-weighted imaging for early detection of cystogenesis and response to therapy in a mouse model of polycystic kidney disease. Kidney International.

[22] Friedli I (2016). New Magnetic Resonance Imaging Index for Renal Fibrosis Assessment: A Comparison between Diffusion-Weighted Imaging and T1 Mapping with Histological Validation. Scientific Reports.

[23] Zhao J (2014). Assessment of renal fibrosis in chronic kidney disease using diffusion-weighted MRI. Clinical Radiology.

[24] Mao W (2018). Intravoxel incoherent motion diffusion-weighted imaging for the assessment of renal fibrosis of chronic kidney disease: A preliminary study. Magnetic Resonance Imaging.

[25] Cai XR (2016). Use of intravoxel incoherent motion MRI to assess renal fibrosis in a rat model of unilateral ureteral obstruction. Journal of Magnetic Resonance Imaging.

[26] Hueper K (2016). Diffusion-Weighted imaging and diffusion tensor imaging detect delayed graft function and correlate with allograft fibrosis in patients early after kidney transplantation. Journal of Magnetic Resonance Imaging.

[27] Xie Y (2018). Functional evaluation of transplanted kidneys with reduced field-of-view diffusion-weighted imaging at 3T. Korean Journal of Radiology.

[28] Feng YZ (2018). Intravoxel incoherent motion (IVIM) at 3.0 T: evaluation of early renal function changes in type 2 diabetic patients. Abdom Radiol (NY).

[29] Liang L (2016). Using intravoxel incoherent motion MR imaging to study the renal pathophysiological process of contrast-induced acute kidney injury in rats: Comparison with conventional DWI and arterial spin labelling. Eur Radiol.

[30] Mao W (2018). Chronic kidney disease: Pathological and functional evaluation with intravoxel incoherent motion diffusion-weighted imaging. J Magn Reson Imaging.

[31] Paul K (2015). Diffusion-sensitized ophthalmic magnetic resonance imaging free of geometric distortion at 3.0 and 7.0 T: a feasibility study in healthy subjects and patients with intraocular masses. Invest Radiol.

[32] Piskunowicz M (2015). A new technique with high reproducibility to estimate renal oxygenation using BOLD-MRI in chronic kidney disease. Magn Reson Imaging.

[33] Milani B (2016). Reduction of cortical oxygenation in chronic kidney disease: evidence obtained with a new analysis method of blood oxygenation level-dependent magnetic resonance imaging. Nephrology Dialysis Transplantation.

[34] Pohlmann A (2013). High temporal resolution parametric MRI monitoring of the initial ischemia/reperfusion phase in experimental acute kidney injury. PLoS One.

[35] Pohlmann A (2017). Experimental MRI Monitoring of Renal Blood Volume Fraction Variations En Route to Renal Magnetic Resonance Oximetry. Tomography.

[36] Norris DG (1991). Ultrafast low-angle RARE: U-FLARE. Magn Reson Med.

[37] Norris DG, Bornert P, Reese T, Leibfritz D (1992). On the application of ultra-fast RARE experiments. Magn Reson Med.

[38] Norris DG, Bornert P (1993). Coherence and Interference in Ultrafast RARE Experiments. Journal of Magnetic Resonance.

[39] Niendorf T, Norris DG, Leibfritz D (1994). Detection of apparent restricted diffusion in healthy rat brain at short diffusion times. Magnetic Resonance in Medicine.

[40] Niendorf T, Dijkhuizen RM, Norris DG, Van Lookeren Campagne M, Nicolay K (1996). Biexponential diffusion attenuation in various states of brain tissue: Implications for diffusion-weighted imaging. Magnetic Resonance in Medicine.

[41] Niendorf T (1999). On the application of susceptibility-weighted ultra-fast low-angle RARE experiments in functional MR imaging. Magn Reson Med.

[42] Heinrichs U (2009). Myocardial T_2_^*^ mapping free of distortion using susceptibility-weighted fast spin-echo imaging: a feasibility study at 1.5 T and 3.0 T. Magn Reson Med.

[43] Utting Jane F., Kozerke Sebastian, Luechinger Roger, Schnitker Ralph, Vohn René, Bhanniny Rani, Tilbian Maral, Niendorf Thoralf (2009). Feasibility of k-t BLAST For BOLD fMRI With a Spin-Echo Based Acquisition at 3 T and 7 T. Investigative Radiology.

[44] Hennig J, Nauerth A, Friedburg H (1986). RARE imaging: a fast imaging method for clinical MR. Magn Reson Med.

[45] Jin N (2011). Targeted single-shot methods for diffusion-weighted imaging in the kidneys. J Magn Reson Imaging.

[46] Hilbert F (2017). Comparison of Turbo Spin Echo and Echo Planar Imaging for intravoxel incoherent motion and diffusion tensor imaging of the kidney at 3Tesla. Z Med Phys.

[47] Williams CFM, Redpath TW, Norris DG (1999). A novel fast split-echo multi-shot diffusion-weighted MRI method using navigator echoes. Magnetic Resonance in Medicine.

[48] Stejskal EO, Tanner JE (1965). Spin Diffusion Measurements: Spin Echoes in the Presence of a Time‐Dependent Field Gradient. The Journal of Chemical Physics.

[49] Alsop DC (1997). Phase insensitive preparation of single-shot RARE: application to diffusion imaging in humans. Magn Reson Med.

[50] Pohlmann A (2014). Detailing the relation between renal T2* and renal tissue pO2 using an integrated approach of parametric magnetic resonance imaging and invasive physiological measurements. Invest Radiol.

[51] Notohamiprodjo M (2015). Combined intravoxel incoherent motion and diffusion tensor imaging of renal diffusion and flow anisotropy. Magn Reson Med.

[52] Zhang X, Ingo C, Teeuwisse WM, Chen Z, van Osch MJP (2018). Comparison of perfusion signal acquired by arterial spin labeling-prepared intravoxel incoherent motion (IVIM) MRI and conventional IVIM MRI to unravel the origin of the IVIM signal. Magn Reson Med.

[53] Zhang B (2018). Application of noninvasive functional imaging to monitor the progressive changes in kidney diffusion and perfusion in contrast-induced acute kidney injury rats at 3.0 T. Abdom Radiol (NY).

[54] Friedli I (2017). Comparison of readout-segmented and conventional single-shot for echo-planar diffusion-weighted imaging in the assessment of kidney interstitial fibrosis. J Magn Reson Imaging.

[55] Friedli I (2015). Improvement of renal diffusion-weighted magnetic resonance imaging with readout-segmented echo-planar imaging at 3T. Magn Reson Imaging.

[56] Kim SG, Hu X, Adriany G, Uğurbil K (1996). Fast interleaved echo-planar imaging with navigator: high resolution anatomic and functional images at 4 Tesla. Magn Reson Med.

[57] Jezzard P (2012). Correction of geometric distortion in fMRI data. Neuroimage.

[58] Holdsworth SJ (2008). Readout-segmented EPI for rapid high resolution diffusion imaging at 3T. European Journal of Radiology.

[59] Porter DA, Heidemann RM (2009). High resolution diffusion-weighted imaging using readout-segmented echo-planar imaging, parallel imaging and a two-dimensional navigator-based reacquisition. Magn Reson Med.

[60] Heidemann RM (2010). Diffusion imaging in humans at 7T using readout-segmented EPI and GRAPPA. Magn Reson Med.

[61] Hillenbrand C, Hahn D, Haase A, Jakob PM (2000). MR CAT scan: a modular approach for hybrid imaging. MAGMA.

[62] Jakob P, Hillenbrand CM, Kenn W, Hahn D, Haase A (2002). Abdominal imaging with a modular combination of spin and gradient echoes. Magnetic Resonance in Medicine: An Official Journal of the International Society for Magnetic Resonance in Medicine.

[63] Chen N-kuei (2013). A robust multi-shot scan strategy for high-resolution diffusion weighted MRI enabled by multiplexed sensitivity-encoding (MUSE). Neuroimage.

[64] Hong X, Thomas Dixon W (1992). Measuring diffusion in inhomogeneous systems in imaging mode using antisymmetric sensitizing gradients. Journal of Magnetic Resonance.

[65] Reese TG, Heid O, Weisskoff RM, Wedeen VJ (2003). Reduction of eddy-current-induced distortion in diffusion MRI using a twice-refocused spin echo. Magn Reson Med.

[66] Shehata M (2018). 3D kidney segmentation from abdominal diffusion MRI using an appearance-guided deformable boundary. PloS one.

[67] Deoni P (2004). Quantitative diffusion imaging with steady-state free precession. Magnetic Resonance in Medicine.

[68] Landolt, Zahlenwerte und Funktionen aus Physik, Chemie, Astronomie, Geophysik und Technik. Berlin Heidelberg New York: Springer-Verlag (1969).

[69] Como G (2008). Relevance of b -values in evaluating liver fibrosis: A study in healthy and cirrhotic subjects using two single-shot spin-echo echo-planar diffusion-weighted sequences. Journal of Magnetic Resonance Imaging.

[70] Schick F (1997). SPLICE: Sub-second diffusion-sensitive MR imaging using a modified fast spin-echo acquisition mode. Magnetic Resonance in Medicine.

