# Supplementary material for: Diffusion-weighted Renal MRI at 9.4 Tesla Using RARE to Improve Anatomical Integrity
Source: Sci Rep. 2019 Dec 23;9:19723. doi: 10.1038/s41598-019-56184-6 (PMC6928203; doi:10.1038/s41598-019-56184-6)
Supplement: Supplementary file 1 — Supplementary Information [file 41598_2019_56184_MOESM1_ESM.docx]

Diffusion-weighted Renal MRI at 9.4 Tesla Using RARE to Improve Anatomical Integrity

Joāo dos Santos Periquito, Katharina Paul, Till Huelnhagen, Min-Chi Ku, Yiyi Ji, Kathleen Cantow, Thomas Gladytz, Dirk Grosenick, Bert Flemming, Erdmann Seeliger, Sonia Waczies, Thoralf Niendorf, Andreas Pohlmann

SUPPLEMENTARY INFORMATION

# Pulse Sequence Diagram


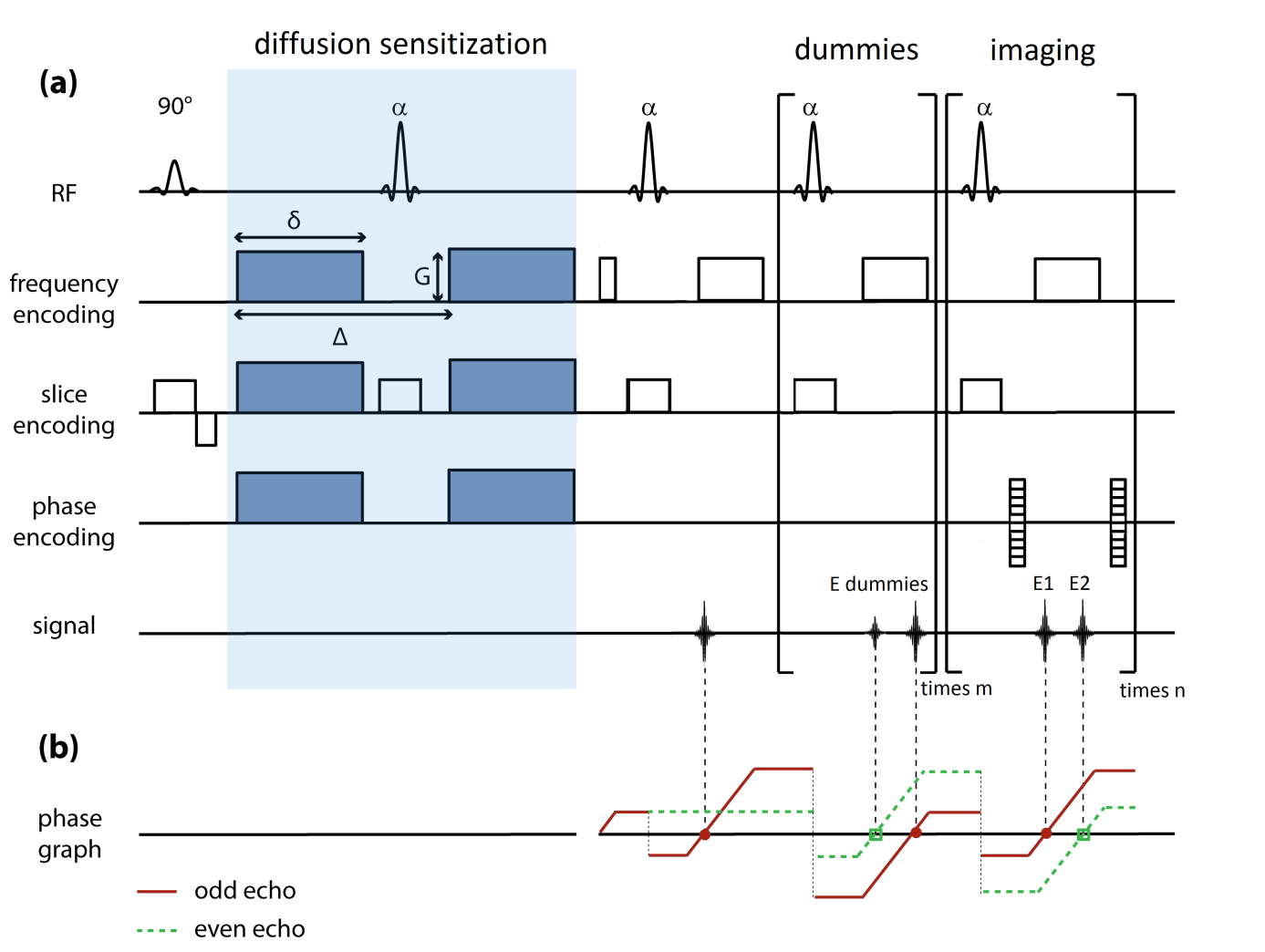


**Pulse Sequence Diagram.** a): Basic scheme of the diffusion-sensitized split-echo RARE sequence ^44^ showing the RF pulse train, the frequency encoding, phase encoding and the slice selection gradients. The dephasing frequency encoding gradient prior to the second refocusing RF pulse α is unbalanced, i.e. the 0th moment of this gradient does not equal half the 0th moment of the readout gradient ^70^. Unipolar diffusion sensitization gradients of amplitude G and duration δ, separated by the delay between the onset of the gradient pulses (Δ), surround the first refocusing pulse in phase, frequency and slice encoding. After the third refocusing pulse, the odd (E1) and the even (E2) echo are generated for the first time. The odd (E1) and the even (E2) echoes are acquired within each echo spacing, reconstructed separately, with the resulting images being added afterwards. (b): The evolution of the magnetization is illustrated in the extended phase graph. Only the two pathways contributing to the signal are shown: the odd echo (red solid line) and the even echo (green dashed line).
